# Responding to AIDS, Tuberculosis, Malaria, and Emerging Infectious Diseases in Burma: Dilemmas of Policy and Practice

**DOI:** 10.1371/journal.pmed.0030393

**Published:** 2006-10-10

**Authors:** Chris Beyrer, Voravit Suwanvanichkij, Luke C Mullany, Adam K Richards, Nicole Franck, Aaron Samuels, Thomas J Lee

## Abstract

Beyrer and colleagues discuss infectious disease threats in Burma and suggest policy options for responding to them.

In 2004 the Global Fund to Fight AIDS, Tuberculosis, and Malaria (“Global Fund”) awarded program grants to Burma (Myanmar) totaling US$98.4 million over five years—recognizing the severity of Burma’s HIV/AIDS and tuberculosis (TB) epidemics, and noting that malaria was the leading cause of morbidity and mortality, and the leading killer of children under five years old [1]. For those individuals working in health in Burma, these grants were welcome, indeed [2].

In that same year, Burma’s authoritarian military regime—the State Peace and Development Council (SPDC)—was accused of severe and ongoing human rights violations, and United Nations Secretary General Kofi Annan appointed a Special Rapportuer on Human Rights, signaling a high level of concern about the junta’s governance. Given these occurrences, the Global Fund imposed additional safeguards on their Burma grants—including additional monitoring of activities and expenditures—and requested and received written guarantees from the junta to respect the fund’s safeguards and performance-based grant system.

On August 18, 2005, the Global Fund announced termination of the grants, stating that “given new restrictions recently imposed by the government which contravene earlier written assurances it has provided the Global Fund, the Global Fund has now concluded that the grants cannot be implemented in a way that ensures effective program implementation” [1]. The fund made it clear that the decision was due to the SPDC having imposed restrictions on access to project implementation areas, and having added additional procedures to the procurement of medical supplies. Despite the statement from the Global Fund, some individuals involved in health within Burma have argued that political pressure from the United States played a role in the withdrawal.

In the same month, the executive director of the World Food Programme visited Burma and called for a relaxation of government controls on the procurement and distribution of food commodities [3]. The World Food Programme reported that one in three Burmese children was chronically malnourished or stunted, and that 15% of the 2005 population of 53 million was food-insecure [3]. In December 2005, the medical charity Médecins Sans Frontières France reported that they, too, were withdrawing from Burma, and again cited junta restrictions on staff travel to project areas as the primary cause [4]. And in February 2006, the International Committee of the Red Cross suspended a prisoner-visiting program as a result of insistence by junta that the junta supervise such visits [5].

In each case, restrictions on health and/or humanitarian activity have included lengthy delays for approval to travel to project sites, variously reported as taking up to three weeks; the addition of junta approved “minders” on all site visits; and limits on the time allowed at sites. In February 2006, the SPDC Ministry of National Planning and Economic Development put forward guidelines for NGOs, UN agencies, and international organizations working in Burma [6]. The guidelines formalize, reaffirm, and expand those restrictions that led to the Global Fund pullout—such as ministry-level approval of programs; coordination, project implementation, opening and registration of field offices; appointment of staff; internal travel; management and equipment purchases; and coordination at the state, division, and township levels [6].

The program withdrawals from Burma highlight what has become a contentious and complex dilemma for the international community: how best to respond to the health crises affecting the people of Burma. While some donors have withdrawn from Burma, others including the European Commission have called for increased levels of humanitarian assistance [7]. And a number of longstanding collaborative groups—such as Population Services International, which has done condom social marketing in Burma since 1996—have continued operations in the country (http://www.psi.org). The aid dilemma has become more compelling as Burma’s health and humanitarian crises have been increasingly recognized as playing important roles in South and Southeast Asia [8].

## Methods

We reviewed the recent medical and policy literature on Burma, with a focus on HIV/AIDS, TB, and malaria. We searched the PubMed database using MeSH terms “Burma” and “Myanmar.” We reviewed all articles from January 1, 2004, to May 1, 2006, relevant to HIV/AIDS, TB, and malaria. We reviewed data available on Burma’s health expenditures and infrastructure and on SPDC policies, and analyzed information from a variety of sources and informants—attempting, wherever possible, to assess the validity of reports. Given the range of limitations on the availability of information from Burma, including restrictions imposed by the SPDC on publications and reports, we acknowledge that there are likely to have been information sources that were unavailable to us. We had the following objectives: attempt to synthesize what is known about HIV/ AIDS, TB, malaria, and other disease threats—including avian influenza (H5N1 virus) in Burma; assess the regional health and security concerns associated with these epidemics; and suggest policy options that would respond to these threats in the context of the tightening restrictions imposed by the junta. Field investigations were conducted by our group and by Burmese colleagues in 2005 and 2006, which included visits to health facilities in Burma and along its borders, as well as discussions with health professionals inside and outside the country.

## SPDC Health Expenditures and Policies

The SPDC has been cited for its markedly low levels of public funding for health and education [8]. Public-sector investment in education and health care combined, in Burma, is less than US$1 per person, per year—one of the lowest levels worldwide [8]. This contributed to Burma’s low ranking in the World Health Organization (WHO) millennium assessment of health-care systems—190th out of 191 nation states [9]. Health and education systems receive less than 3% and 10% of expenditures, respectively [10,11]. Table 1 shows recent disease-specific expenditures for SPDC budgets. The 2003 malaria expenditure figure of US$23 million is the largest disease-specific expenditure reported that year by the Burmese government. The entire health budget for that year was US$18 million in 2003, meaning that this malaria figure must be interpreted with caution.

**Table 1:**
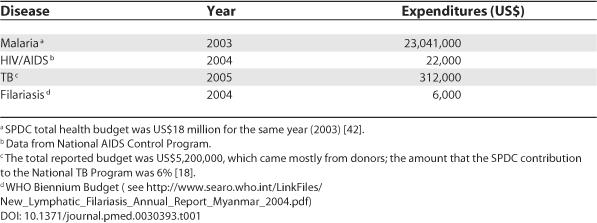
SPDC Expenditures for Disease Control, 2003–2005, for Selected Infectious Diseases

Widespread corruption has weakened the health sector [11–14]. Disinvestment has also eroded Burma’s educational system, a situation that continues to worsen as many skilled educators and professionals have emigrated [15]. Where available in Burma, libraries and other educational facilities are still inadequate [16,17]. Although the junta purports to be producing more graduates, decreases in medical and nursing education standards raise questions about the skills of recent graduates (http://www.myanmar.com/Ministry/health/g09.htm).

Burma’s laboratory infrastructure has also weakened, both directly as a result of disinvestment and indirectly from creating a dearth of skilled technical personnel [18]. The US Centers for Disease Control and Prevention mission to Burma is one of the few external laboratory assessments available. The mission’s assessment was that laboratory infrastructure in the public sector was markedly underfunded and underdeveloped, and in need of expansive investment [19].

## HIV/AIDS, TB, and Malaria in Burma

### HIV/AIDS.

We reported an estimate of the scale of Burma’s HIV/AIDS epidemic in 2003 using data from the national HIV sentinel surveillance and a national household survey to generate conservative estimates of HIV prevalence [2]. We estimated a population rate of 3.46% of reproductive age adults living with HIV in mid-year 2000, with a range of 2.72%–4.19% [2]. The Joint UN Programme on HIV/AIDS estimated a slightly lower prevalence in 2000 and some 46,000 AIDS-related deaths that year—Burma’s national reporting system identified 802 AIDS-related deaths [2]. The sentinel surveillance was apparently either suspended or not reported until March–April 2003, when it was again undertaken (Tables 2 and 3).

**Table 2:**
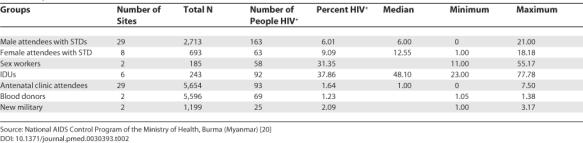
HIV Sentinel Surveillance Data from March–April, 2003, from the National AIDS Control Program of the Ministry of Health, Burma (Myanmar)

**Table 3:**
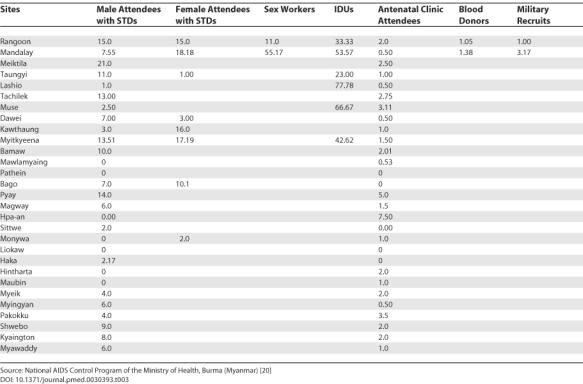
HIV Seropositivity (%) by Sites, among Sentinel Populations, March–April, 2003

Table 2 shows the 2003 national HIV sentinel surveillance results. Most risk groups were sampled only in Rangoon and Mandalay. Overall, sample sizes were small, making the ranges of minimum and maximum prevalence (shown as reported by the National AIDS Control Programme) quite wide: the median HIV prevalence among the 2,713 male patients with sexually transmitted diseases (STDs) surveyed was 6%, giving a minimum of 0% and a maximum of 21%. The minimum would suggest low HIV prevalence; the maximum, the highest in Asia. Similarly, the range for pregnant women is 0%–7.5% HIV prevalence—wide enough to make interpretation difficult. These data suggest that the HIV sentinel surveillance is probably too limited in scale and scope to accurately capture HIV/AIDS in this large and diverse country [20].

Table 3 presents the same 2003 surveillance data by site; gives percentages, not absolute numbers; and suggests additional concerns with the surveillance system. Sex worker rates in the two cities sampled are quite divergent, with 11% reported prevalence in Rangoon and 55.17% in Mandalay. Looking at rates among pregnant women from the same two cities, we see 2.0% prevalence in Rangoon, but strikingly lower HIV seropositivity (0.50%) in Mandalay. While such an outcome is possible given the small samples, it is unlikely. Further uncertainty is evidenced as women attending an STD clinic in Rangoon had higher rates of HIV/AIDS than sex workers, while the opposite was true in Mandalay. Such divergent findings suggest more methodologic and sampling differences than true variance in rates. The US Centers for Disease Control and Prevention mission to Burma in 2002 came to the same conclusion, advocating for improved surveillance methods and laboratory upgrades [21]. The Centers for Disease Control and Prevention also proposed a collaboration to make these improvements. This effort did not go forward when the junta refused to allow for confidential voluntary counseling and testing [21, 22]. All HIV-positive test results continue to be reported to the junta.

Molecular epidemiologic data from several groups working both within the country and in the country’s border regions with India, China, and Thailand suggest that Burma’s epidemic is characterized by marked viral diversity, high rates of recombination and circulating recombinant forms, and high rates of HIV and hepatitis C coinfection among injection drug users (IDUs) [22–30]. This molecular picture has been linked to Burmese heroin-trafficking routes, and to the very-high-exposure settings among IDUs in upper Burma. IDUs typically use drugs at tea-stall settings—where injection equipment is kept on the premises and is used repeatedly by multiple users, creating opportunities for viral interactions. Despite some declines in heroin production and export, Burma remains the world’s second-leading producer and the largest heroin exporter in Southeast Asia [29,30].

### TB.

South and Southeast Asia have the highest burden of TB worldwide: one in three cases of TB diagnosed worldwide is in this region [31]. Burma reports some 97,000 new cases diagnosed each year to WHO, which WHO has suggested may be underreporting the actual incidence [1,32–34]. Overall, approximately 40% of Burma’s population is estimated to be infected with TB [34]. The Ministry of Health has designated TB as a priority disease [18]. Nevertheless, Burma has been rated by WHO as moving far too slowly to adequately control TB—a problem identified by WHO as far back as 1998 as being due to a lack of political will and commitment [32].

The epidemic of TB in Burma is closely linked to HIV. WHO estimates that approximately 6.8% of patients with TB in Burma have HIV, while 60%–80% of patients living with HIV also have TB—making TB the most common opportunistic infection in people living with HIV/AIDS [31,34,35] Burma has the highest mortality rate—i.e., 2.8 per 100,000—among patients with TB who are coinfected with HIV in Southeast Asia [32].

With the introduction of WHO’s Directly Observed Therapy, Short-Course (DOTS) program in Burma in 1997, the government reports that case detection rates have improved [32]. In 2005 the country claims to have 100% DOTS coverage among its 324 townships and a treatment success rate of 81%—just below WHO’s goal of 85% for 2005 [19,31,36]. But program data suggest that this optimistic scenario may not be accurate. A 2005 WHO report noted that “the National TB Program (NTP) budget was around US$3 million in 2002, but a large funding gap meant that actual expenditures were only around US$1 million, primarily for staff and first-line drugs” [19]. It also noted a “…shortage of qualified staff, especially junior laboratory technicians,” and that “…a quarter of all sanctioned posts in the NTP are vacant” [19].

There is also microbiologic evidence—i.e., rising antibiotic resistance—that the TB control program in Burma is troubled. In the first report on multidrug-resistant (MDR) TB in Burma, in 2000, 33.3% of isolates from patients newly diagnosed with pulmonary TB were resistant to at least one first-line drug, and 2% of isolates were MDR TB [37]. In a 2005 follow-up analysis by the same group, 33.9% of TB isolates were resistant to any one of the four standard first-line drugs, with the rate of MDR TB more than doubling to 4.2%; among patients who had received treatment in the past, this figure rose to 18.4% [38]. The authors found that a history of exposure to TB treatment for at least one month was associated with over 3-fold odds of developing MDR TB. Average MDR rates in Southeast Asia are roughly 2.0%, so Burma’s official MDR-TB rates are more than double those of neighboring countries [39]. One report from the Thai side of the Thai–Burma border found that MDR TB accounted for 6.5% of TB isolates, compared with 0.9% for the rest of Thailand [40].

### Malaria.

Burmese official statistics reported to WHO show that malaria causes proportionately more mortality in Burma than in any other country in Southeast Asia [41]. Burma reported over 700,000 cases of clinically suspected malaria in 2004, of which almost 80% was Plasmodium falciparum, and consistently records the most malaria-related deaths of any country in the region [41–43]. This figure puts Burma’s reported malaria caseload at about 7.3% of the WHO regional disease burden, but responsible for 53.6% of malaria-related deaths in the region—many of which occur in children under five (Figure 1) [1,41,42].

**Figure 1:**
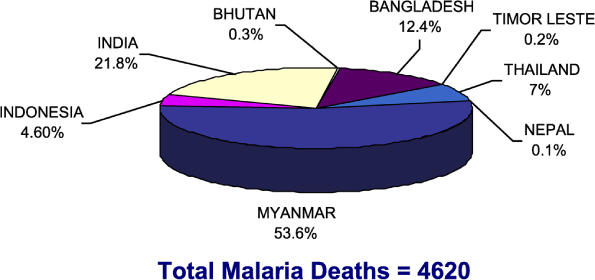
Distribution of Reported Malaria Deaths in Southeast Asia, 2003 Burma accounts for about 7% of malaria cases reported in Southeast Asia, including India, yet over half the malaria deaths in the region occur in this country. Source: WHO, SEAR [75]

According to 2003 WHO data, the annualized country-wide incidence rate of malaria is approximately 3.6 cases per 1,000 population, (0.36%) per year [41]. The areas at greatest risk are the forested border areas of Burma, populated by ethnic-minority groups living in areas marked by civil conflict (Figure 2). Over half of Burma’s cases of malaria are reported from just 100 townships in these areas, which account for only 25% of the population (13.7 million people) [44]. In one study, the malaria-related morbidity was highest in the Chin and Karenni states, located on the Indian and Thai borders, respectively [45]. Kachin state on the Chinese border, another ethnic-minority region, had mortality rates for malaria almost five times higher than the national average [46]. Among internally displaced persons in the conflict zones of eastern Burma, malaria accounted for 45% of all adult and child deaths in 2003–2004 [47,48].

**Figure 2:**
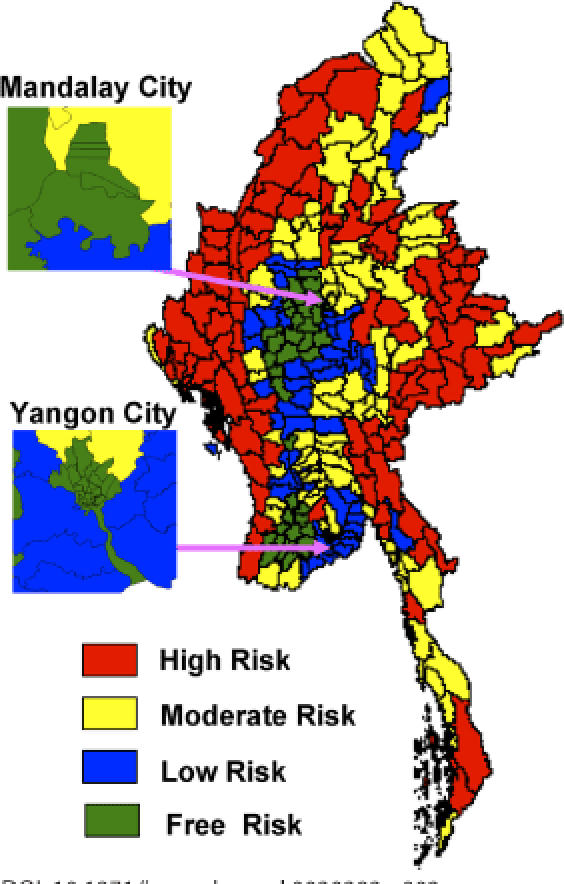
Malaria Risk Areas in Burma The areas at highest risk are overwhelmingly along the frontiers, where civil confl ict is ongoing and widespread human rights abuses have been documented. Source: WHO Regional Offi ce for South-East Asia [76]

Burma has also become an epicenter for drug-resistant P. falciparum, particularly along the frontiers of the country [45]. Chloroquine and sulfadoxine-pyrimethamine (SP), two former mainstays of malaria treatment, are too frequently ineffective if used alone, so they have been abandoned in favor of combination therapies in the region [45,49–51]. The Thai–Burma border has documented significant levels of clinical treatment failure and in vitro resistance against quinine and mefloquine: between 1986 and 1997, a 10-fold decrease in mefloquine sensitivity was noted, and the use of this drug alone is also no longer effective [45,50].

MDR P. falciparum malaria, defined as malaria that is resistant to three or more drugs, is most problematic on the Thai–Burma border [45]. As a result, combination therapy with artesunate and high-dose mefloquine is now the recommended treatment for falciparum malaria. There are currently no other practical alternatives in this area to artemisinin compounds [45,52]

Several factors may hasten the spread of drug-resistant malaria, including incomplete or inappropriate use of antimalarials, program failure, and the sale and use of fake or expired antimalarials. There is little regulatory oversight of the importation and sale of antimalarials in Burma, and the proportion of fake drugs is high [53]. Dondorp et al. reported in 2004 that 21% of artesunate tested in Burma contained no active ingredients [54].

The more expensive artemesinin derivatives strain already scarce resources available for treatment of malaria, and many Burmese continue to purchase their medications over the counter and on the black market [44,45,52,54]. Counterfeit artesunate, containing little or no active compound, is now common in Burma, where over one-fifth of drugs sampled in one analysis were fake [53–55].

## Other Diseases and Health Threats: Avian Flu

Avian flu is a regional and global concern. Given Burma’s poor health and laboratory infrastructure and the limits on access to Burma, the concern over Burma’s potential to aid in the evolution of avian flu is prudent. Burma’s first report of the H5N1 virus describes emergence on March 8, 2006, on a farm in the Mandalay district, about 430 miles north of Rangoon [56].

Some 112 birds died in the outbreak. Veterinary authorities in the country called for international assistance, but the state-run media did not notify the Burmese people of the threat until March 17, 2006, more than a week later [57]. Six days after junta officials first began to investigate bird deaths and three days after the Ministry of Livestock and Fisheries confirmed the presence of H5N1, Burma’s state-run press was silent on the issue. By March 18, 2006, more that 10,000 birds were reported to have died, and 41,000 birds culled [57].

The main concern with the failure to inform the public about avian flu is the risk of further unwitting spread (through continued transport and sale of animals, or through other exposures to animal waste). Such spread has subsequently been reported in Burma by the UN Food and Agriculture Organization (FAO), with over 100 bird flu outbreaks reported by FAO at an April 10, 2006, press conference in Bangkok [58]. He Changchui, the FAO representative for Asia-Pacific, stated that “the issue there is that awareness is rather poor. The information is not that comprehensive” [58]. Encouragingly, specimens from Mandalay were sent to Bangkok for confirmatory testing, and the SPDC was reportedly cooperating with the FAO: they quarantined poultry farms around the initial outbreak, and they have shared samples from infected animals with Thailand and Australia [57]. The delay in alerting the Burmese people, however, appears to have undermined the effectiveness of the early response.

## Regional Issues and Security Threats

How important are Burma’s ongoing epidemics of HIV/AIDS, TB, and malaria to the country’s neighbors? A driver of regional importance in HIV/AIDS has been the interaction between the spread of HIV and narcotics use and exports from Burma. Burma remains the world’s second-largest opium- and heroin-producing state, and has dramatically increased its production and export of methamphetamine [29,59]. In Burma’s border regions with China and India, the epidemiologic impact of Burma’s HIV/AIDS epidemic has been driven on both sides of the country’s borders by spread related to illicit drug use [60]. A recent report also adds the Burma–Bangladesh border to those regions where heroin exports from Burma have led to increases in heroin use [61].

Yunnan Province, in China’s southwest, is the area with the highest HIV prevalence in China. Epidemic spread in Yunnan began among IDUs in several districts on the Yunnan–Burma border in the early 1990s [62]. The uptake of heroin use, and subsequent epidemics of IDU-related infections, including HIV and hepatitis C, are direct outcomes of Burma’s heroin exports to China [62,63].

The National AIDS Control Program of India, in collaboration with the Avahan Program, has mapped the HIV prevalence rates among pregnant women across India’s HIV- surveillance-reporting districts [64]. These data show high rates of infection in two of India’s most remote regions, the northeastern states of Manipur and Nagaland—both of which border Burma. Both states are on principal overland heroin-trafficking routes out of Burma [60].

The population exodus from Burma has made MDR TB an issue of regional concern [16]. Isolates collected from patients on the Thai side of the border who are Burmese migrants reveal that MDR TB now accounts for 6.5% of TB isolates, compared with the national average of 0.9% for the rest of Thailand [40]. In some Thai provinces bordering Burma, almost half of all patients with TB are not Thai, and cure rates among these individuals is low—25.8% in one analysis—threatening Thailand’s ability to control TB and raising costs [65,66].

The large-scale migration of Burmese people across international borders has significant regional malaria-control implications, especially for Thailand, where malaria incidence rates have been steadily declining. This declining trend is not seen in those provinces that share a border with Burma [67,68]. Of these, Tak Province, adjacent to Burma’s Karen state, has the highest numbers of cases of malaria, and in one study the prevalence of asymptomatic parasitemia in Tak migrants was over 20 times that of the local Thai population (4.4% versus 0.2%) [69].

MDR-malaria parasites are common along the Thai–Burma border [43]. A similar spillover effect has also been noted in India and China, where migration across porous borders threatens to undermine local malaria-control efforts [69–73]. On India’s northeastern border with Burma, the risk of treatment failure decreases with increasing distance away from the border [73].

## Discussion and Conclusion

Given the scale and scope of Burma’s health needs, are there policy options for health work in this current climate? Several options for working in Burma may have some utility: (1) cross-border approaches, (2) use of independent media for health education and information, and (3) expanded support through international organizations and NGOs (nongovernmental organizations).

One of our findings was that much of the available data on HIV/AIDS, TB, and malaria come from border areas. Indeed, one might assume that the health threats themselves were most marked in the Thai–Burma border areas. However, at least some of the emphasis on data from this area is no doubt a result of the paucity of information from areas bordering India, Bangladesh, the Lao People’s Democratic Republic, and China. Cross-border efforts, where feasible, are likely to increase in importance in regard to the health of the Burmese people if SPDC restrictions continue to tighten.

Many communities of internally displaced people on the Thai–Burma border rely on a network of mobile indigenous health workers, known collectively as “Backpack Health Worker Teams,” for basic health care. These communities have very poor access to health services and are considered inhabitants of “black zone,” or “conflict” areas, by the SPDC. These health workers are indigenous, highly mobile, and have successfully navigated the ongoing conflict to provide much-needed care and information.

A number of other ethnic groups within Burma have health organizations that operate clinics and/ or mobile backpack teams (including the Arakanese, Chin, Kachin, Shan, Palaung, Karenni, and Mon) on all of Burma’s borders, with varying degrees of development. The Karen team, for example, runs more than 30 clinics, servicing a population of over 80,000; the Kachin, more than 60 clinics, servicing a population of 98,000 [74]. With international support, these ethnic groups could have the potential to reach a substantial proportion of the population with two key characteristics: (1) the highest disease burden and (2) the highest potential to transmit infection to neighboring countries.

The new government guidelines for the provision of humanitarian assistance put forth by the Ministry of National Planning and Economic Development in early 2006, if enforced, is likely to markedly diminish independent capacity to respond to health threats in Burma. Nevertheless, a number of organizations and NGOs will continue their activities in Burma, as well as continue to provide needed services. While a range of donors are engaged in efforts to counter the funding losses incurred by the pullout of the Global Fund, the new restrictions are more explicitly supportive of junta control of activities, staffing, site visits, procurement, and the like. The fundamental dilemma of how best to respond to health threats and provide humanitarian assistance while the SPDC further restricts access to Burma’s neediest has worsened in 2006.

Health-related programs such as Population Services International, Médecins Sans Frontières Netherlands, and numerous others that are currently functioning in Burma are likely to continue. But from a public health perspective, much more fundamental and widespread change will be required to actually meet the scale and scope of Burma’s HIV/AIDS, TB, and malaria epidemics, increasing malnutrition, and other health threats. While such reform in Burma has been painfully slow, disease spread can be markedly rapid. Perhaps the concerns over emergent avian flu will have more regional impact than the tragedies of HIV/AIDS, TB, and malaria in Burma have had to date.

*A longer version of this report is available at http://www.jhsph.edu/humanrights/research. The Chinese- and Burmese-language versions of the executive summary are available at http://www.soros.org/initiatives/bpsai/articles_publications/publications/respondingtoaids_20060330.*

## Abbreviations

DOTS: directly observed therapy, short-course
FAO: Food and Agriculture Organization
IDU: injection drug user
MDR: multidrug resistant
NGO: nongovernmental organization
SPDC: State Peace and Development Council
STD: sexually transmitted disease
TB: tuberculosis
WHO: World Health Organization
